# Cadmium Induces Apoptosis in Pancreatic β-Cells through a Mitochondria-Dependent Pathway: The Role of Oxidative Stress-Mediated c-Jun N-Terminal Kinase Activation

**DOI:** 10.1371/journal.pone.0054374

**Published:** 2013-02-06

**Authors:** Kai-Chih Chang, Ching-Cheng Hsu, Shing-Hwa Liu, Chin-Chuan Su, Cheng-Chieh Yen, Ming-Jye Lee, Kuo-Liang Chen, Tsung-Jung Ho, Dong-Zong Hung, Chin-Ching Wu, Tien-Hui Lu, Yi-Chang Su, Ya-Wen Chen, Chun-Fa Huang

**Affiliations:** 1 Department of Physiology and Graduate Institute of Basic Medical Science, School of Medicine, College of Medicine, China Medical University, Taichung, Taiwan; 2 Institute of Toxicology, College of Medicine, National Taiwan University, Taipei, Taiwan; 3 Department of Otorhinolaryngology, Head and Neck Surgery, Changhua Christian Hospital, Changhua County, Taiwan; 4 Department of Occupational Safety and Health, College of Health Care and Management, Chung Shan Medical University, Taichung, Taiwan; 5 Department of Occupational Medicine, Chung Shan Medical University Hospital, Taichung, Taiwan; 6 Department of Surgery, Peng-Hu Hospital, Makung City, Taiwan; 7 Department of Health, Executive Yuan, Taipei, Taiwan; 8 Department of Urology, China Medical University Hospital, Taichung, Taiwan; 9 School of Chinese Medicine, College of Chinese Medicine, China Medical University, Taichung, Taiwan; 10 Division of Toxicology, Trauma & Emergency Center, China Medical University Hospital, Taichung, Taiwan; 11 Department of Public Health, China Medical University, Taichung, Taiwan; Juntendo University School of Medicine, Japan

## Abstract

Cadmium (Cd), one of well-known highly toxic environmental and industrial pollutants, causes a number of adverse health effects and diseases in humans. The growing epidemiological studies have suggested a possible link between Cd exposure and diabetes mellitus (DM). However, the toxicological effects and underlying mechanisms of Cd-induced pancreatic β-cell injury are still unknown. In this study, we found that Cd significantly decreased cell viability, and increased sub-G1 hypodiploid cells and annexin V-Cy3 binding in pancreatic β-cell-derived RIN-m5F cells. Cd also increased intracellular reactive oxygen species (ROS) generation and malondialdehyde (MDA) production and induced mitochondrial dysfunction (the loss of mitochondrial membrane potential (MMP) and the increase of cytosolic cytochrome *c* release), the decreased *Bcl-2* expression, increased *p53* expression, poly (ADP-ribose) polymerase (PARP) cleavage, and caspase cascades, which accompanied with intracellular Cd accumulation. Pretreatment with the antioxidant *N*-acetylcysteine (NAC) effectively reversed these Cd-induced events. Furthermore, exposure to Cd induced the phosphorylations of c-jun N-terminal kinases (JNK), extracellular signal-regulated kinases (ERK)1/2, and p38-mitogen-activated protein kinase (MAPK), which was prevented by NAC. Additionally, the specific JNK inhibitor SP600125 or JNK-specific small interference RNA (si-RNA) transfection suppressed Cd-induced β-cell apoptosis and related signals, but not ERK1/2 and p38-MAPK inhibitors (PD98059 and SB203580) did not. However, the JNK inhibitor or JNK-specific si-RNA did not suppress ROS generation in Cd-treated cells. These results indicate that Cd induces pancreatic β-cell death via an oxidative stress downstream-mediated JNK activation-triggered mitochondria-regulated apoptotic pathway.

## Introduction

Cadmium (Cd) is a naturally occurring nonessential toxic heavy metal that is widely distributed in the earth's crust. It has many industrial applications, e.g., use in several alloys, color pigments, electroplating, and rechargeable nickel-cadmium batteries [1]. In mammals, the major sources of Cd exposure are contaminated water and food, cigarette smoke, and industrial pollutants [2]–[3]. Cd content in human can gradually increase by exposure to Cd-contaminated foods, which is through bio-accumulated effects raising Cd levels in the food chain (such as 0.06 mg/L vs. 0.42–0.63 mg/L of Cd in rice from non-vs. Cd-contaminated areas), and may cause a higher incidence of many Cd-related diseases, including renal dysfunction, hepatotoxicity, osteoporosis, and cancers [4]–[6]. Although Cd preferentially accumulates in the liver, kidney, bone, and the pancreas is also an important target organ [1], [4], [7]. The pancreas is a unique organ that is composed of 4 different cell types, including alpha, beta, delta, and pancreatic polypeptide producing (PP) cells, and located within the endocrine part of the tissue [8]. Animal studies have shown that Cd can cause pancreatic β-cell damage, suppress insulin secretion, increase glucose intolerance, and have diabetogenic effects [9]–[10]. In sub- or chronic investigations, a marked disturbance of glucose homeostasis, the destruction of pancreatic islets insulin secretion, the increase in gluconeogenic enzymes, and even significant enhance Cd toxicity in diabetic conditions are observed after rats exposed to Cd (0.5–2 mg/kg/day), which are accompanied with significant Cd accumulation in the blood and/or pancreas [9], [11]–[13]. Moreover, epidemiological studies reveal significantly higher blood and urine levels of Cd in DM patients compared to healthy individuals [14]–[17]. Long-term exposure to Cd results in a marked increase in blood glucoses and decrease in plasma insulin secretion that are associated with blood and urinary levels of Cd [13], [18]. Some *in vivo* studies have indicated a possible link between Cd exposure and diabetogenic effects (blood glucose unbalance and pancreatic islets dysfunction); however, the precise mechanisms of Cd-induced toxicological effects on the function of pancreatic β-cells and injuries remain unclear.

Oxidative stress is a key risk factor for many undesirable biological reactions and functional cell damage, and it has been implicated in a wide variety of disease processes, including neurodegenerative diseases and diabetes mellitus (DM) [19]. Pancreatic β-cells are susceptible to oxidative stress damage, which is generated by chronic exposure to high levels of glucose and toxic agents causing pancreatic β-cell dysfunction and apoptosis [20]–[22]. It has been reported that oxidative stress can activate members of mitogen-activated protein kinase (MAPK) cascades, including JNK, ERK, and p38 MAPK. These proteins mediate many important signals in mammal cells, including cell proliferation, differentiation, and apoptosis [23]–[24]. Activation of MAPK pathways may result in either control cell survival or apoptosis depending on the stimuli [25]. Recently, a number of studies reported that oxidative stress contributes to both the onset and the progression of DM, which is correlated with JNK- or p38 MAPK-regulated pathways [26]–[28].

Growing evidence indicates that heavy metals such as mercury, arsenic, and nickel induce impairments in pancreatic β-cell function through the induction of oxidative stress [21], [29]. Toxic metals induce excessive amounts of reactive oxygen species (ROS) eliciting oxidative stress damage that results in many pathophysiological processes and development of disease [21], [30]. It has also been documented that Cd-induced toxic effects are closely associated with the production of ROS, which can destroy DNA, proteins, and lipid function, and activate signaling pathways that cause cell death [31]–[32]. Both oxidative stress damage and the activation of MAPK pathways are associated with Cd-induced apoptosis. In a variety of cell types, including skin epidermal and neuronal cells, Cd-induced oxidative stress damage not only activates JNK, ERK1/2, and p38, but also triggers p53- or mTOR-mediated signals that cause apoptosis [33]–[34]. However, the role of oxidative stress in Cd-induced activation of MAPK signals regulated apoptosis in pancreatic β-cells has not yet been studied. The aim of this study is to investigate the cytotoxicity of Cd in the pancreatic β-cells and to elucidate the cellular mechanism involved in the Cd-induced β-cell apoptosis.

## Materials and Methods

### Materials

Unless specified, otherwise, all chemicals (including CdCl_2_) and laboratory plastic wares were purchased from Sigma-Aldrich (St. Louis, MO, USA) and Falcon Labware (Bectone-Diskinson, Franlin Lakes, NJ, USA), respectively. RPIM 1640 medium, fetal bovine serum (FBS), and antibiotics were purchased from Gibco (Gibco BRL, Life Technologies, USA). Insulin antiserum immunoassay kit was supplied from (Mercodia (Mercodia AB, Sweden). 2′, 7′-dichlorofluorescin diacetate (DCFH-DA), and 3,3′-dihexyloxacarbocyanine iodide (DiOC**_6_**) were purchases from Molecular Probes (Eugene, OR, USA). Commercial LPO assay kit was purchased from Calbiochem (San Diego, USA). Mouse- or rabbit-monoclonal antibodies specific for cytochrome c, JNK-1, ERK1/2, p38, α-tubulin, and secondary antibodies (goat anti-mouse or anti-rabbit IgG-conjugated horseradish peroxidase (HRP)) were purchased fron Santa Cruz Biotechnology (Santa Cruz Biotechnology, Inc., CA, USA), and PARP, caspase-3, caspase-7, caspase-9, phosphor (p)-JNK, p-ERK1/2, and p-p38 were purchased from Cell Signaling Technology (Cell Signaling Technology, Inc., Danvers, MA, USA).

### Pancreatic β-cell-derived RIN-m5F cell culture

RIN-m5F cell is a rat pancreatic β-cell line, showing the production and secretion of insulin. Cells were purchased from American Type Culture Collection (ATCC, CRL-11605), and cultured in a humidified chamber with a 5% CO_2_-95% air mixture at 37°C and maintained in RPIM 1640 medium supplemented with 10% fetal bovine serum (FBS) and antibiotics (100 U/mL of penicillin and 100 μg/mL of streptomycin).

### Measurement of cell viability

Cells were washed with fresh media and cultured in 96-well plates (2×10^4^/well) and then stimulated with CdCl_2_ (1–10 μM) for 24 h. After incubation, the medium was aspirated and cells were incubated with fresh medium containing 0.2 mg/mL 3-(4, 5-dimethyl thiazol-2-yl-)-2, 5-diphenyl tetrazolium bromide (MTT) was added. After 4 h, the medium was removed and the blue formazan crystals were dissolved in 100 μL dimethyl sulfoxide (DMSO). Absorbance at 570 nm was measured using an enzyme-linked immunosorbent assay (ELISA) microplate reader (Bio-tek µQuant Monochromatic Microplate Spectrophometer, MTX Lab Systems, Inc.).

### Determination of insulin secretion

To measure the amount of insulin secretion in RIN-m5F cells after exposure to CdCl_2_, cells were performed in Krebs Ringer buffer (KRB) under 20 mM glucose-stimulated condition, and supernatant of cmedia was immediately collected and stored at −20°C, as previously described [29]. To measure the amount of insulin secretion, aliquots of samples were collected from the plasma or experimental solutions at indicated time points, and subjected to insulin antiserum immunoassay kit according to the manufacturer's instructions.

### Determination of malondialdehyde (MDA) formation

The formation of MDA, a substance produced during lipid peroxidation (LPO), was determined by using the commercial LPO assay kit [35]. Briefly, RIN-m5F cells were seeded at 1×10^6^ cells/well in a 6-well plate and incubated with CdCl_2_ in the absence or present (1 h pre-treatment) of NAC for 24 h. At the end of treatment, cells were harvested and homogenized in 20 mM Tris-HCl buffer, pH 7.4, containing 0.5 mM butylated hydroxytoluene to prevent sample oxidation. The equal volumes samples (cell supernatant or plasma) were assayed immediately using the lipid peroxidation assay kit. An absorbance of 586 nm was measured using an ELISA microplate reader. The protein concentration was determined using the bicinchoninic acid protein assay kit with an absorption band of 570 nm. LPO level was expressed as nanomoles (nmol) MDA per milligram protein and estimated from the standard curve.

### Measurement of ROS production

Intracellular ROS generation was monitored by flow cytometry using the peroxide-sensitive fluorescent probe: 2′, 7′-dichlorofluorescin diacetate (DCFH-DA). In brief, RIN-m5F cells were seeded at 2×10^5^ cells/well in a 24-well plate and incubated with CdCl_2_ in the absence or present (1 h pre-treatment) of NAC. At the end of various time course treatments, cells were incubated with medium containing 20 μM DCFH-DA for 15 min at 37°C. After incubation with the dye, cells were harvested and washed twice with PBS, and then re-suspended in ice-cold phosphate buffered saline (PBS) and placed on ice in a dark environment. The intracellular peroxide levels were measured by flow cytometer (FACScalibur, Becton Dickinson, Sunnyvale, CA, USA), that emitted a fluorescent signal at 525 nm.

### Determination of mitochondrial membrane potential (MMP)

MMP was analyzed using the fluorescent probe DiOC_6_, with a positive charge of a mitochondria-specific fluorophore. Briefly, RIN-m5F cells were seeded and incubated with CdCl_2_ in the same manner as for ‘Measurement of ROS production’ analysis for 8 h. At the end of treatment, cells were incubated with medium with 100 nM 3,3′-dihexyloxacarbocyanine iodide (DiOC_6_) for 30 min at 37°C. After incubation with the dye, cells were harvested and washed twice with PBS, and then re-suspended in ice-cold phosphate buffered saline (PBS). MMP was analyzed by a flow cytometer (excitation at 475 nm and emission at 525 nm; FACScalibur, Becton Dickinson).

### Measurement of sub-G1 DNA content

RIN-m5F cells were seeded and incubated with CdCl_2_ in the same manner as for ‘Measurement of ROS production’ analysis for 24 h. At the end of treatment, cells were harvested and washed twice with PBS, and then re-suspended in 1 mL of cold 70% (v/v) ethanol and stored at 4°C for 24 h. Then, the fixed cells were washed twice with PBS and incubated at 37°C for 30 min with 1 mg/mL RNase-A dissolved in 0.5 mL of 0.2% Triton X-100/PBS solution. Following the incubation, cells were washed with PBS, the cells were stained with 50 µg/mL propidium iodide (PI) at 4°C for 30 min in dark conditions. The stained cells were subjected to flow cytometry analysis of DNA content (FACScalibur, Becton Dickinson). Nuclei displaying hypodiploid, sub-G1 DNA contents were identified as apoptotic.

### Detection of apoptotic cells

Apoptosis was assessed using Annexin V fluorescent probe, a protein that binds to phosphatidylserine (PS) residues which are exposed on the cell surface of apoptotic cells. Cells were treated with or without CdCl_2_ for 24 h. After treatment, cells were washed twice with PBS (PH 7.4), and incubated with Annexin V-Cy3 (Ann Cy3) and 6-carboxy fluorescein diacetate (6-CFDA) simultaneously (Annexin V-Cy3^TM^ apoptosis detection kit). After labeled at room temperature, cells were immediately observed by fluorescence microscopy (Axiovert 200, Zeiss, 200x). Ann Cy3 was available for binding to PS, which was observed as red fluorescence. In addition, cell viability could be measured by 6-CFDA, which was hydrolyzed to 6-CF and appears as green fluorescence. Cells in the early stage of apoptosis would be labeled with both Ann Cy3 (red) and 6-CF (green).

### Determination of cadmium content

RIN-m5F cells were seeded at 5×10^6^ cells/dish in a 6 cm^2^ dish and treated with CdCl_2_ for different time interval. After incubation, Cells were harvested and washed with PBS three times followed by addition of 0.15% nitric acid, and the mixture was vortexed and frozen at −20°C for 2 h or overnight. Tubes were thawed at 37°C for 20 mins and centrifuged at 1,000× g at 4°C for 10 mins. The supernatant (the intracellular cadmium levels) was determined by inductively coupled plasma mass spectrometry (ICP-MS). The detection limit for cadmium was ∼0.1 ppb(ug/L).

### Western blot analysis

Equal amounts of proteins (50 μg per lane) was subjected to electrophoresis on 10% (W/V) SDS-polyacrylamide gels and transferred to polyvinylidene difluoride (PVDF) membranes. The membranes were blocked for 1 h in PBST (PBS, 0.05% Tween-20) containing 5% nonfat dry milk. After blocking, the membranes were incubated with rabbit anti-rat antibodies against cytochrome c, PARP, caspase-3, caspase-7, caspase-9, p-JNK, p-p38, p-ERK1/2, JNK-1, ERK1/2, p38, or α-tubulin in 0.1% PBST (1∶1000) for 1 h at room temperature. After they were washed in 0.1% PBST followed by two washes (15 min each), the blots were subsequently incubated with goat anti-mouse or anti-rabbit IgG-HRP secondary antibody (1∶1000) for 1 h. The antibody-reactive bands were revealed by enhanced chemiluminescence substrate (Perkin-Elmer^TM^, Life Sciences) and were exposed on the Fuji radiographic film.

### Real-time quantitative reverse-transcribed polymerase chain reaction (RT-PCR) analysis

The expression of apoptosis-related genes was evaluated by real-time quantitative RT-PCR (qPCR) analysis that carried out using Taqman® one-step PCR Master Mix (Applied Biosystems, Foster City CA). Briefly, total cDNA (100 ng) was added per 25-μL reaction with sequence-specific primers and Taqman® probes. Sequences for all target gene primers and probes were purchased commercially (*β*-actin, the housekeeping gene, was used as an internal control) (Applied Biosystems, CA). Quantitative RT-PCR assays were carried out in triplicate on StepOnePlus sequence detection system. Cycling conditions were 10 min of polymerase activation at 95°C followed by 40 cycles at 95°C for 15 s and 60°C for 60 s. After 40 cycles, samples were run on a 2% agarose gel to confirm specificity. Data analysis was performed using StepOne^TM^ software (Version 2.1, Applied Biosystems). All amplification curves were analyzed with a normalized reporter (R_n_: the ratio of the fluorescence emission intensity to the fluorescence signal of the passive reference dye) threshold of 0.2 to obtain the C_T_ values (threshold cycle). The reference control genes were measured with four replicates in each PCR run, and their average C_T_ was used for relative quantification analyses (the relative quantification method utilizing real-time PCR efficiencies [36]). TF expression data were normalized by subtracting the mean of reference gene C_T_ value from their C_T_ value (ΔC_T_). The Fold Change value was calculated using the expression 2^−ΔΔC^_T_, where ΔΔC_T_ represents ΔC_T-condition of interest_ – ΔC_T-control_. Prior to conducting statistical analyses, the fold change from the mean of the control group was calculated for each individual sample.

### Small interference RNA transfection

Specific small interference-RNA (si-RNA) against rat JNK and control si-RNA were purchased commercially from Cell Signaling Technology. RIN-m5F cells were seeded in 6- or 24-well culture plates and transfected with the si-RNA using Lipofectamine RNAi MAX (Gibco/Invitrogen) according to the manufacturer's instructions. Cellular levels of the proteins specific for the si-RNA transfection were checked by Western blot, and all experiments were performed at 24 h after transfection.

### Animal preparation

We purchased normal male ICR mice (4 weeks old, 20–25 g) from the Animal Center of College of Medical, National Taiwan University. The Animal Research Committee of China Medical University conducted the study in accordance with the guidelines for the care and use of laboratory animals. The protocols were approved by the Committee on the ethics of Animal Experiments of China Medical University (Permit Number: 98-30-N). Mice were housed in a room at a constant temperature (23±2°C), 50±20% relative humidity, given a solid diet and tap water ad-libidum, and 12 hrs of light-ark cycles. Mice were acclimatized to the laboratory conditions prior to the experiments and all experiments were carried out between 8:00 AM and 05:00 PM. Mice were administered CdCl_2_ (0.5 and 1 mg/kg/day, oral application by gavage) for 6 consecutive weeks in the presence or absence of NAC (150 mg/kg). Each group was contained 15 mice (*n* = 15). All experimental mice were sacrificed by decapitation under pentobarbital anesthesia (80 mg/kg, i.p.), and the whole blood samples were collected from the peripheral vessels. Whole blood sample were centrifuged at 3000× *g* for 10 min, then plasma was obtained, and insulin and LPO levels were assayed immediately. At the same time, pancreas were quickly removed and stored at liquid nitrogen until use.

### Oral glucose tolerance test (OGTT)

Oral glucose tolerance testing was performed as previously described (Chen et al., 2006). Mice were administrated with D-glucose (1 g/kg) by stomach tube after an overnight fast. Blood samples were collected before and 30, 60, 90, and 120 min after delivery of the glucose load. Blood glucose levels were determined using the Bayer blood glucose meter (Ascensia ELITE, Bayer).

### Apoptosis analysis in mouse islet

Islet cells apoptosis was detected by TUNEL method utilizing the Dead End Colorimetic Apoptosis Detection system (Promega Corporation, Pty. Ltd., USA). The deep brown of TUNEL-positive cells was imaged under the Nikon ECLIPSE 80i upright microscope equipped with a charge-coupled device camera (with ×400 magnification).

### Statistical analysis

Data are presented as means ± standard deviations (S.D.) The significance of difference was evaluated by the Student's t-test. When more than one group was compared with one control, significance was evaluated according to one-way analysis of variance (ANOVA) was used for analysis, and the Duncans's post hoc test was applied to identify group differences. The P value less than 0.05 was considered to be significant. The statistical package SPSS, version 11.0 for Windows (SPSS Inc., Chicago, IL, USA) was used for the statistical analysis.

## Results

### Cd affected cell viability, insulin secretion, and ROS generation in pancreatic β-cells

**Figure 1:**
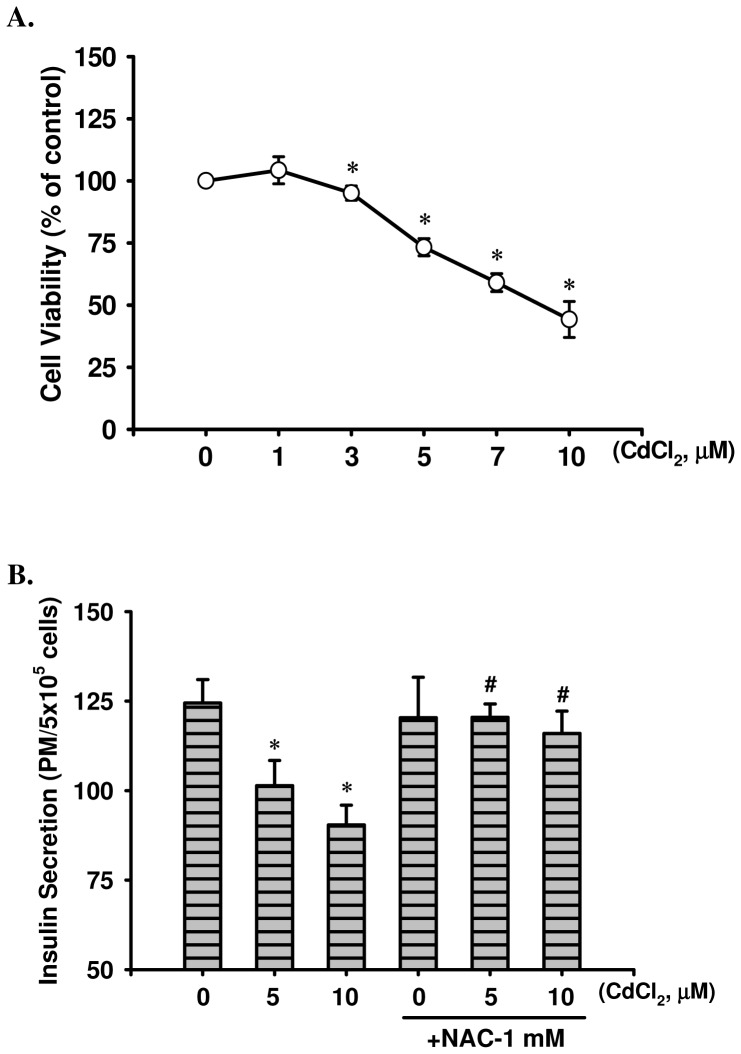
Effects of Cd on cytotoxicity and insulin secretion in RIN-m5F cells. (A) Cells were treated with or without CdCl_2_ (1–10 μM) for 24 h, and cell viability was determined by MTT assay. (B) Insulin secretion from RIN-m5F cells with or without CdCl_2_ (5 and 10 μM) in the absence or presence of NAC (1 mM) for 4 h was detected under 20 mM glucose-stimulated condition. Data are expressed as mean ± S.D. for six independent experiments with triplicate determination. *p<0.05 as compared with vehicle control. **^#^** *p*<0.05 as compared with cadmium alone.

To investigate Cd-induced pancreatic β-cell cytotoxicity, cell viability was determined in rat pancreatic β-cell-derived RIN-m5F cells using the MTT assay. Cell viability of RIN-m5F cells was significantly reduced in a dose-dependent manner after exposure to 3 to 10 μM CdCl_2_ for 24 h (Figure 1A). Moreover, to evaluate the effect of Cd-induced the dysfunction of insulin secretion, the short-term response of Cd on the insulin secretion from β-cells was detected. After 4 h of treatment (that was not induced the reduction of viable cells), Cd (5 and 10 μM) effectively suppressed insulin secretion in RIN-mF cells (5 uM CdCl_2_, 101.37±7.06 pM; 10 uM CdCl_2_, 90.43±5.48 pM; control, 124.48±6.55 pM, *n* = 6, *p<0.05)(Figure 1B). In addition, treatment of male ICR mice with CdCl_2_ (0.5 and 1.0 mg/kg/day, that mimics the possible human exposed dosage and route [48]) over a 1- to 6-weeks period displayed a slight but significant depression in body weight gain relative to the vehicle control (Figure S1), caused a marked decrease in plasma insulin levels (Cd-0.5 mg/kg group, 142.88–95.23 pmol/L; Cd-1.0 mg/kg group, 125.91–75.08 pmol/L; age-matched control group, 172.99–169.33 pmol/L; *n* = 15, *p<0.05)(Figure S2–A), and glucose intolerance appeared an elevation and plasma insulin levels after glucose loading for 30 min observed a decrease in mice exposed to Cd for 6 consecutive weeks (Figure S2–B), which was accompanied with the significant accumulation of Cd in the whole blood (Cd-0.5 mg/kg group, 1.31±0.11–2.98±0.56 μg/L; Cd-1.0 mg/kg group, 3.64±0.49–9.46±2.45 μg/L; age-matched control group, 0.25±0.05–0.14±0.05 μg/L, *n* = 15, *p<0.05)(Table S1) and in the pancreas (Cd-0.5 mg/kg group, 26.80±3.76–149.0±14.8 ng/g w.t.; Cd-1.0 mg/kg group, 78.40±12.7–339.0±19.4 ng/g w.t.; age-matched control group, 2.33±0.57–5.33±0.51 ng/g w.t., *n* = 15, *p<0.05)(Table S2).

**Figure 2:**
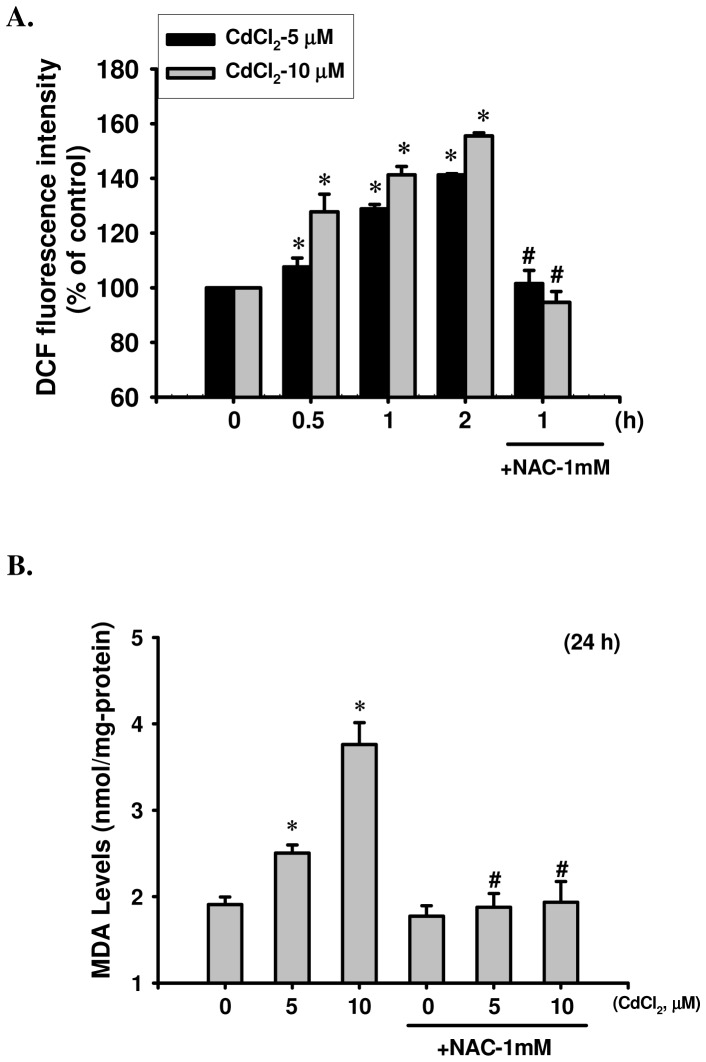
Effects of Cd on oxidative stress damage in RIN-m5F cells. Cells were exposed to CdCl_2_ (5 and 10 μM) for various time courses in the absence or presence of NAC (1 mM), and (A) ROS generation was determined by flow cytometry, and (B) oxidative damage to membrane lipid (lipid peroxidation) was measured the levels of MDA as described in the Materials and Methods section. All data are shown as mean ± S.D. for six independent experiments with triplicate determination. *p<0.05 as compared with vehicle control. **^#^** *p*<0.05 as compared with cadmium alone.

To examine the effect of Cd on the oxidative stress injuries, we performed time-course studies to determine intracellular ROS generation using flow cytometry. Results were showed that exposure of RIN-m5F cells to Cd (5 and 10 μM) resulted in a significant increase in the intensity of dichlorofluorescein (DCF) fluorescence (an indicator of ROS formation) (Figure 2A). Concomitantly, exposure to CdCl_2_ (5 and 10 μM) for 24 h also caused substantial MDA production in the cell membrane after exposure to CdCl_2_ (5 and 10 μM) for 24 h (Figure 2B); and plasma LPO analysis of mice exposed to Cd (0.5 and 1.0 mg/kg/day) over a 1 to 6 weeks period showed that plasma MDA levels dramatically increased in a time-dependent manner (Cd-0.5 mg/kg group 1, 2, 4, and 6 week: 4.79±0.18, 5.00±0.19, 5.49±0.14, and 5.63±0.16 nmole MDA/mg-protein, respectively; Cd-1.0 mg/kg group 1, 2, 4, and 6 week: 4.98±0.17, 5.46±0.13, 6.16±0.20, and 6.33±0.22 nmole MDA/mg-protein, respectively; age-matched control group 1, 2, 4 and 6 week: 4.58±0.11, 4.53±0.15, 4.52±0.11, and 4.53±0.14 nmole MDA/mg-protein, respectively, *n* = 15, *p*<0.05)(Figure S2–C). Co-treatment with an antioxidant NAC effectively blocked Cd-induced toxicological effects of β-cells, including: cytotoxicity (5 μM CdCl_2_, 73.27±3.45%, with NAC, 97.25±2.97% of control; 20 μM CdCl_2_, 44.23±7.25%, with NAC, 99.43±1.93% of control, respectively, *n* = 6, *p*<0.05 as compared with CdCl_2_ alone, data not show), suppressed the insulin secretion (Figure 1B and Figure S2–A), elevated in glucose intolerance (Figure S2–B) and oxidative stress injuries (Figure 2 and Figure S2–C).

### Cd-induced apoptosis is mediated by an oxidative stress-regulated mitochondrial-dependent pathway in RIN-m5F cells

**Figure 3:**
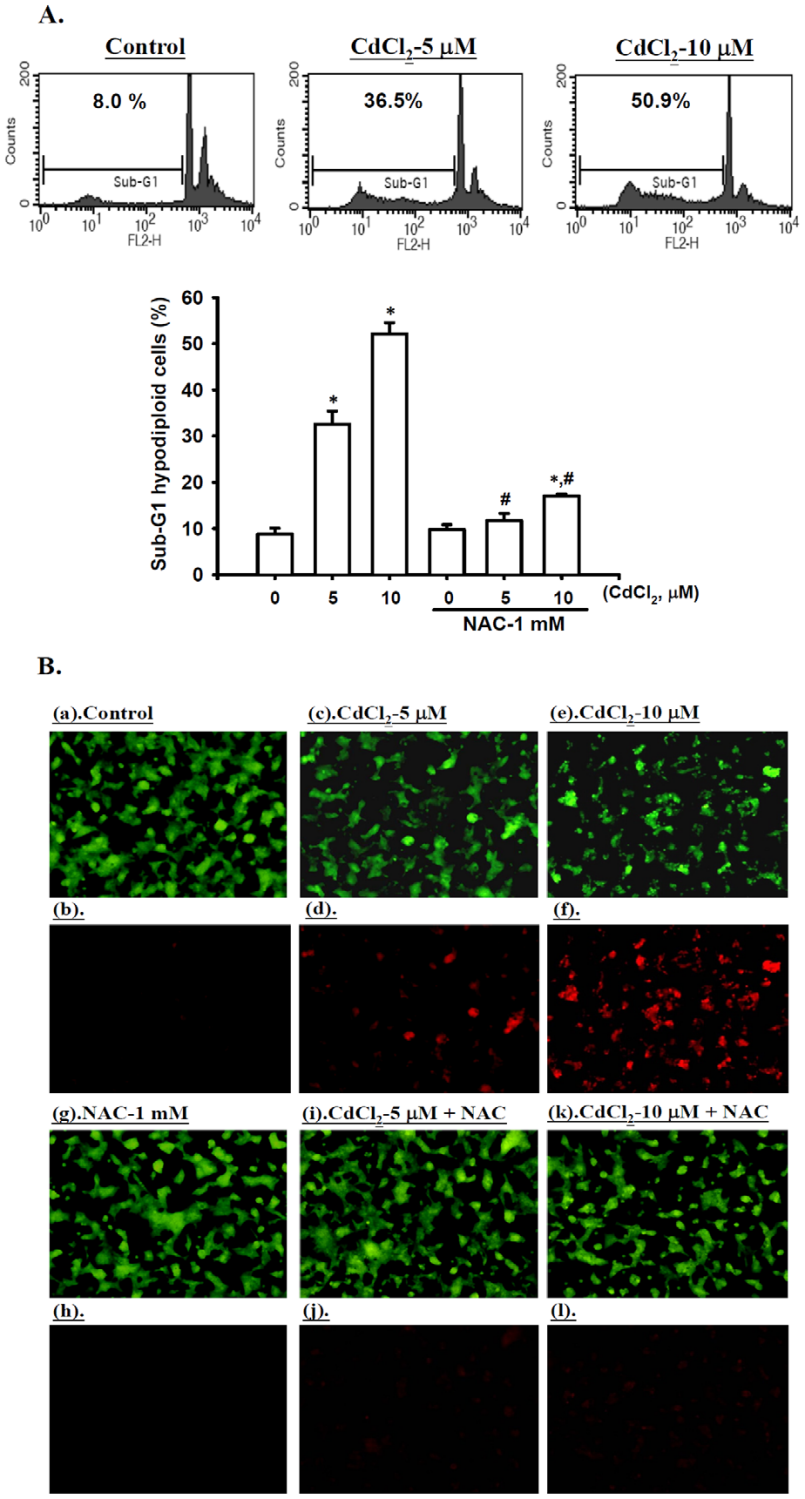
Cd induced apoptosis in RIN-m5F cells. Cells were exposed to CdCl_2_ (5 and 10 μM) for 24 h in the absence or presence of NAC (1 mM), and then processed for (A) genomic DNA fragmentation (sub-G1 DNA content) analysis by flow cytometry, and (B) apoptotic cell observation by Annexin V-Cy3 (red fluorescence) and 6-CFDA (green fluorescence) staining (a and b, control; c and d, CdCl_2_-5 μM; e and f, CdCl_2_-10 μM; g and h, NAC-1 mM; i and j, CdCl_2_-5 μM + NAC; k and l, CdCl_2_-10 μM + NAC) as described in the Materials and Methods section. Results in A are expressed as mean ± S.D. for six independent experiments with triplicate determination after normalizing the result of cell viability. *p<0.05 as compared with vehicle control. **^#^** *p*<0.05 as compared with cadmium alone.

In order to determine whether apoptosis was involved in Cd-induced pancreatic β-cells cytotoxicity, we analyzed the sub-G1 hypodiploid cell population using flow cytometry and determined the level of phosphatidyl serine (PS) the externalization (a hallmark of early apoptotic events) by an annexin V-Cy3/6-CFDA double-staining assay in RIN-m5F cells that were exposed to CdCl_2_ (5 and 10 μM) for 24 h. As shown in Figure 3A, we observed that Cd treatment significantly increased the sub-G1 hypodiploid cell population (genomic DNA fragmentation). Exposure of the cells to Cd also increased the number of cells labeled with both Ann Cy3 (red) and 6-CF (green) fluorescence as compared with untreated cells labeled with green fluorescence (6-CF)(Figure 3B). In addition, it was also revealed the marked trigger in islet cell apoptosis (the deep brown of TUNEL-positive cells) after exposure of mice to Cd (0.5 and 1.0 mg/kg) for 6 consecutive weeks (Figure S2–D). These responses could be effectively abrogated by the treatment with NAC. Therefore, these data indicate that treatment of the pancreatic β-cells with Cd-induced cytotoxicity is dependent on ROS generation that contributes to the apoptosis.

**Figure 4:**
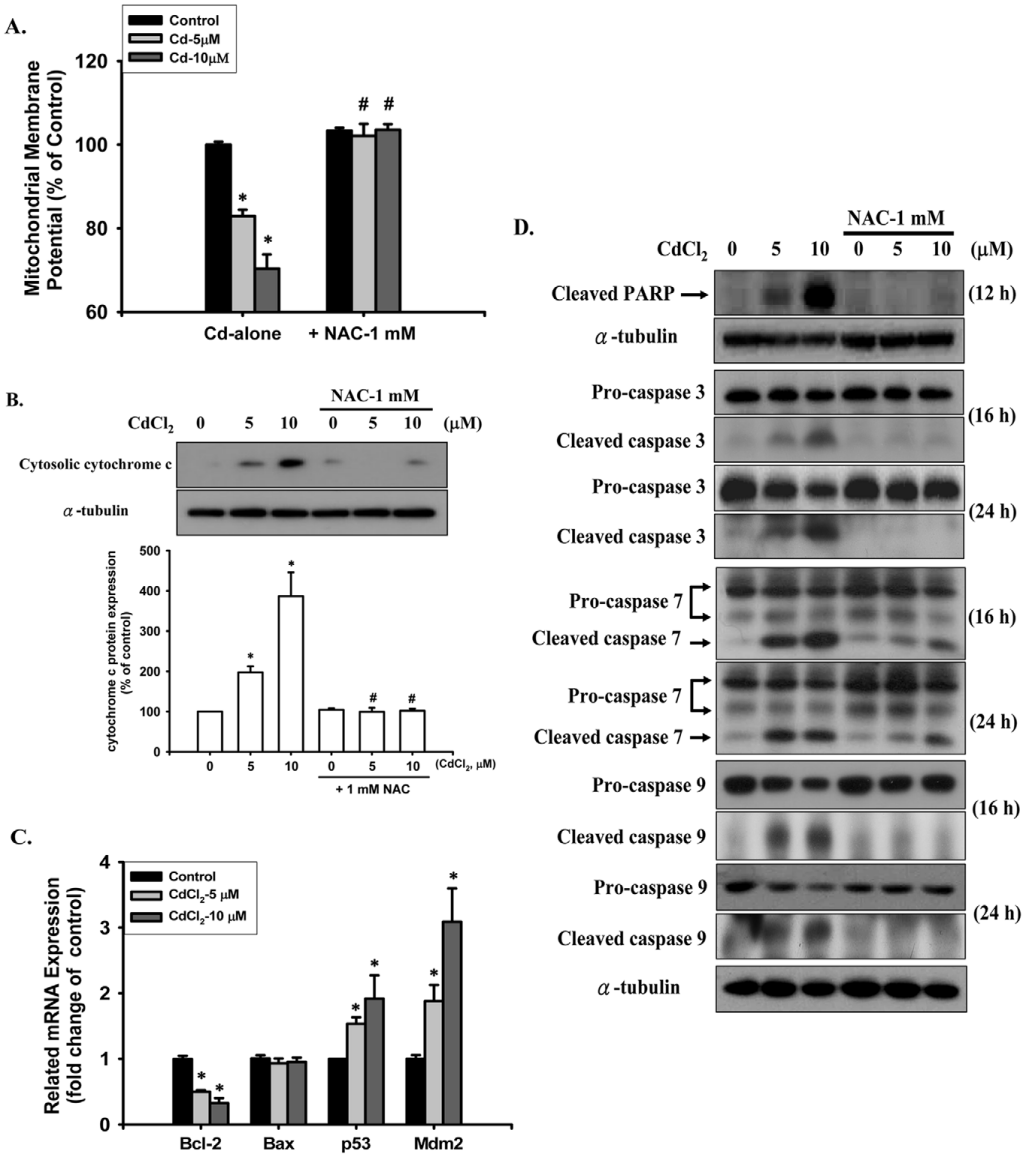
Cd induced mitochondrial dysfunction, poly (ADP-ribose) polymerase (PARP) cleaved and caspase cascades activation in RIN-m5F cells. Cells were exposed to CdCl_2_ (5 and 10 μM) for different time intervals in the absence or presence of NAC (1 mM), and then (A) mitochondrial membrane potential (MMP) depolarization was detected by flow cytometry (for 8 h); (B) cytosolic cytochrome c release was followed by Western blot using the indicated antibody (for 8 h); (C) the expression of anti-apoptotic (*Bcl2* and *Mdm2*) and pro-apoptotic (*Bax* and *p53*) genes was analyzed by quantitative RT-PCR (for 24 h), and (D) PARP cleavage (for 12 h) and caspase-3, -7, and -9 activation (for 16 ad 24 h) were determined by Western blotting analysis as described in the Materials and Methods section. Data in A are shown as mean ± S.D. for six independent experiments with triplicate determination. Results shown in B, D, and E are representative of at last three independent experiments, and α-tubulin was used as loading control. In the experimental of B, a representative data were shown, and the intensity of bands is quantified by densitometric analysis as mean ± S.D. relative control of triplicate experiments after normalizing the bands to α-tubilin. *p<0.05 as compared with vehicle control. **^#^** *p*<0.05 as compared with cadmium alone.

To examine whether Cd-induced apoptosis in the pancreatic β-cells was mediated by a mitochondria-dependent pathway, we determined whether Cd-could modulate the expression of MMP and cytochrome *c*. It was significantly induced the loss of MMP in a dose-dependent manner after exposure of RIN-m5F cells to CdCl_2_ (5 and 10 μM) for 8 h (Figure 4A). Western blot analysis also revealed that exposure of cells to Cd for 8 h markedly increased the release of cytochrome *c* from mitochondria into the cytosolic fraction (Figure 4B). Meanwhile, treatment of RIN-m5F cells with 5 and 10 μM CdCl_2_ for 24 h down-regulated the expression of *Bcl-2* (anti-apoptotic) and up-regulated in *p53* (pro-apoptotic), but did not asffect mRNA levels of *Bax* (Figure 4C). We also found a concomitant increase in *Mdm2* expression, suggesting the induction of a negative regulatory response to the increase in p53 expression. Thus, Cd induced a significant shift in the anti-apoptotic (Bcl-2)/pro-apoptotic (Bax) expression ratio toward a state associated with apoptosis.

**Figure 5:**
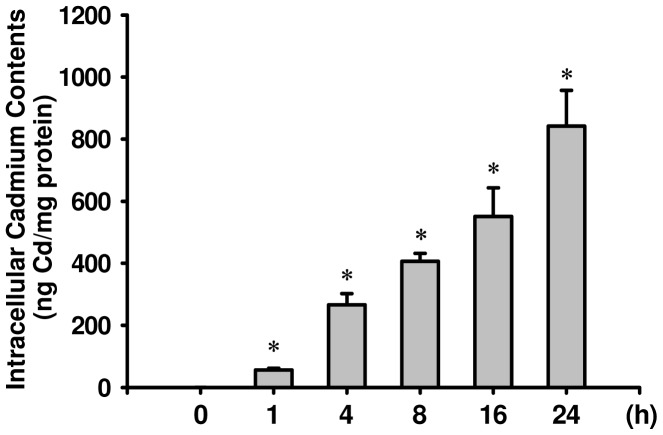
Detection of intracellular Cd content in RIN-m5F cells exposed to CdCl_2_. Cells were exposed to CdCl_2_ (5 and 10 μM) forh, and intracellular Cd content was detected by inductively coupled plasma mass spectrometry (ICP-MS) as described in the Materials and Methods section. Data are shown as mean ± S.D. for six independent experiments with triplicate determination. *p<0.05 as compared with vehicle control.

Furthermore, we also detected the cleavage of PARP and activation of caspase cascade proteases. As shown in Figure 4D, the cleaved form of PARP and significant degradation of full-length pro-caspase-3, pro-caspase-7, and pro-caspase-9 were markedly increased after exposure of RIN-m5F cells to CdCl_2_ (5 and 10 μM) for 12-24 h, which closely corresponded to the accumulation of intracellular Cd levels (Figure 5). These Cd-induced apoptotic responses could be effectively reversed by treatment with NAC (1 mM). These results indicate that Cd-induced pancreatic β-cell death is through a ROS-regulated mitochondria-dependent apoptosis pathway.

### JNK signaling played a critical role in Cd-induced apoptosis of RIN-m5F cells

**Figure 6:**
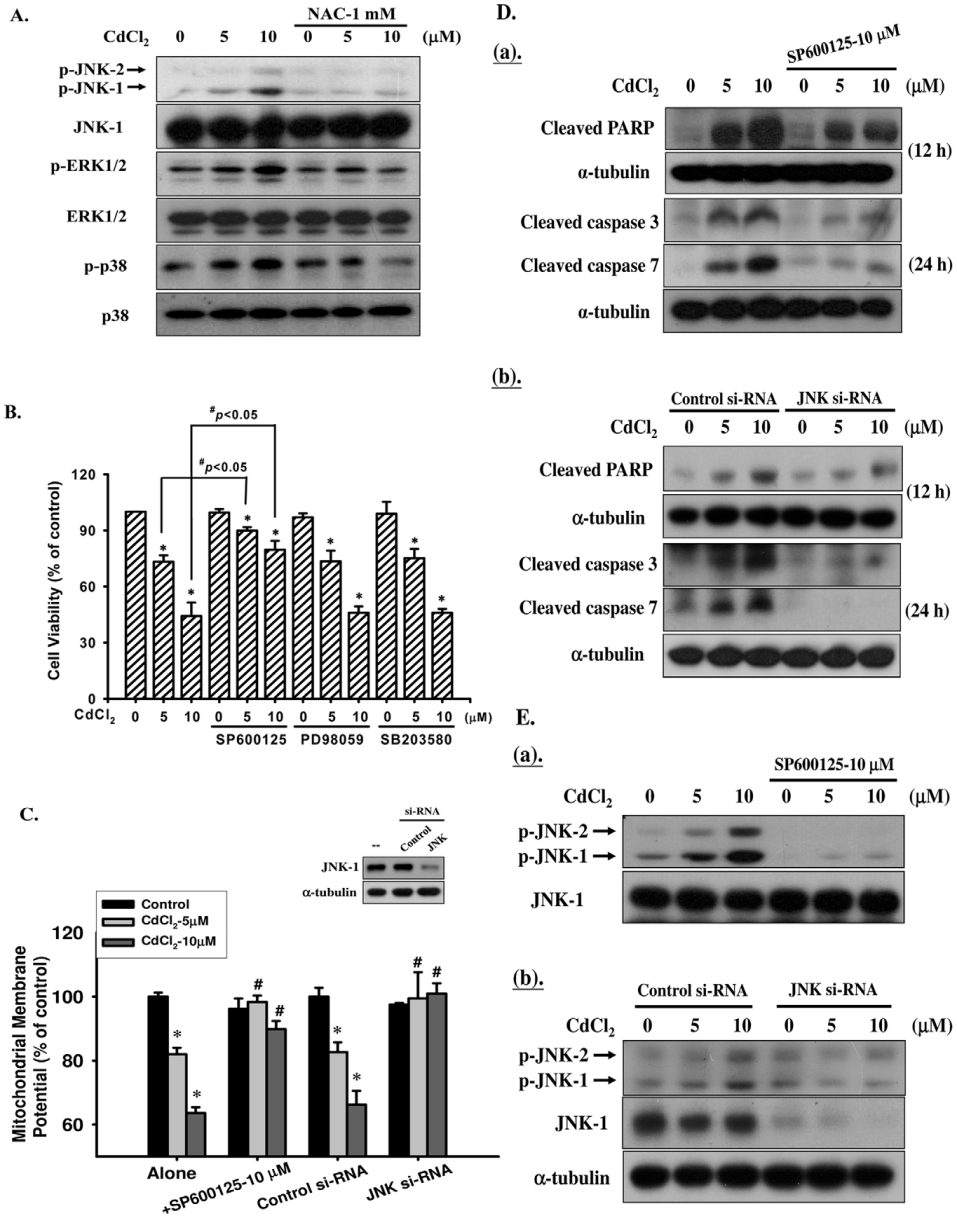
JNK-mediated signaling played a critical role in Cd-induced apoptosis in RIN-m5F cells. (**A**) Cells were incubated with CdCl_2_ (5 and 10 μM) for 0.5 h in the absence or presence of NAC (1 mM), and then analyzed the protein phosphorylation of JNK-, ERK1/2- and p38-MAPK by Western blotting. (B) RIN-m5F cells were exposed to CdCl_2_ in the absence or presence of 10 μM of JNK inhibitor, SP600125, ERK inhibitor, PD98059, or p38 inhibitor, SB203580 for 24 h, and cell viability was determined by MTT assay. In addition, RIN-m5F cells were pre-treated with specific JNK inhibitor (for 1 h) or transfected with si-RNA specific to JNK (following 24 h incubation, JNK-1 expression was examined by Western blot (upper panel of C)) prior to CdCl_2_ (5 and 10 μM) incubation, and MMP depolarization (C, for 8 h) were detected by flow cytometry, PARP cleavage and caspase-3 and -7 activations (D-*a* and D-*b*, for 12–24 h), and the protein phosphorylation of JNK-MAPK (E-*a* and E-*b*, for 0.5 h) were analyzed by Western blot. Results shown in A, D, and E are representative of at last three independent experiments, and α-tubulin was used as loading control. Data in B and **C** are presented as mean ± S.D. for six independent experiments with triplicate determination. *p<0.05 as compared with vehicle control. **^#^** *p*<0.05 as compared with arsenic alone.

MAPK-mediated pathways have critical roles in apoptosis and a variety of stresses, such as ROS and toxic chemicals, can induce MAPK activation. To examine whether MAPK activation was involved in Cd-induced pancreatic β-cell apoptosis, the phosphorylation of MAPK protein was analyzed by Western blot. We found that exposure of RIN-m5F cells to CdCl_2_ (5 and 10 μM) for 30 min resulted in a significant increase in the expression of phosphorylation of JNK, ERK1/2, and p38-MAPK in a dose-dependent manner, which could be reversed by NAC (1 mM) (Figure 6A). Pretreatment of β-cells with the specific JNK inhibitor SP600125 (10 μM), but not that ERK1/2 inhibitor PD98059 or p38-MAPK inhibitor SB203580, for 30 min before exposing them to CdCl_2_ (5 and 10 μM) for 24 h significantly increased the number of viable cells, as compared to those treated with CdCl_2_ alone (Figure 6B). Moreover, pretreatment with JNK inhibitor SP600125 or transfection of cells with JNK-specific si-RNA also prevented loss of MMP, decreased activation of PARP, caspase-3 and caspase-7, and abrogated JNK phosphorylation (Figure 6C–6E), as compared to Cd treatment alone.

**Figure 7:**
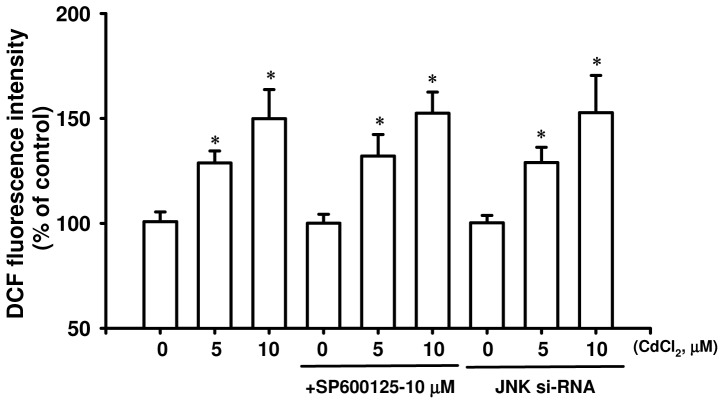
Effects of JNK inhibition on Cd-induced ROS generation in RIN-m5F cells. Cells were pre-treated with specific JNK inhibitor (SP600125-10 μM, for 1 h) or transfected with si-RNA specific to JNK (following 24 h incubation) prior to CdCl_2_ (5 and 10 μM) incubation for 1 h, and ROS was determined by flow cytometry. Data are presented as mean ± S.D. for six independent experiments with triplicate determination. *p<0.05 as compared with vehicle control.

Because Cd induction both of ROS and JNK activation down-regulated the pancreatic β-cell apoptotic signals, we next examined whether there was the relationship between ROS generation and JNK activation in Cd-induced pancreatic β-cell death. Pretreatment with NAC (1 mM) for 30 min followed by exposure to CdCl_2_ (5 and 10 μM) effectively suppressed Cd-induced ROS generation (for 1 h, Figure 2A) and JNK activation (for 30 min, Figure 6A) in RIN-m5F cells. However, pretreatment with SP600125 or transfected with si-RNA specific to JNK did not prevent the Cd-induced ROS production (Figure 7). These findings indicate that ROS-triggered JNK pathway activation in the down-regulated mitochondria-dependent apoptosis play an important role in Cd-induced pancreatic β-cell death.

## Discussion

Cd affects a wide range of cellular events including abnormal proliferation, apoptosis, and carcinogenesis. The cytotoxic effects of Cd in the liver and kidney have been extensively studied, as indicated by apoptotic cell production, formation of nuclear DNA fragmentation, and release of mitochondrial apoptotic proteins after exposure to CdCl_2_, and are accompanied by ROS generation [37]–[38], however, only little studies are focus on the pancreatic β-cells [12]. A major finding of this study, for the first time, demonstrated that Cd induces oxidative stress-activated the JNK pathway, which plays a crucial role in the apoptosis of pancreatic β-cells. Exposure of RIN-m5F cells to Cd stimulated ROS generation and JNK-MAPK activation, which caused the apoptotic-related signals leading to pancreatic β-cell death. The antioxidant NAC and a pharmacological JNK inhibitor (SP600125) or JNK-specific si-RNA transfection effectively prevented Cd-induced phosphorylation of JNK, activation of mitochondrial dysfunction, caspase cascades activation, and apoptosis. However, only NAC suppressed Cd-induced ROS generation. These results demonstrate that Cd induces cell death mainly through the activation of JNK-regulated mitochondria-dependent apoptosis signaling in pancreatic β-cells, and that oxidative stress acts as a pivotal mediator of this signaling.

Oxidative stress, elicited by environmental stimuli and toxic chemicals, has been defined as a disturbance in the pro-oxidant/antioxidant balance that results in functional cell damage, undesirable biological reactions, and implicated in several pathological processes including DM [28], [30]. Pancreatic β-cells, which function in insulin biosynthesis/secretion in mammals, are at greater risk of apoptosis due to ROS attack than other cell types. The mitochondria of β-cells can generate excessive levels of ROS and are both the major source of ROS in these cells and also a primary target for ROS attack. This, combined with a failure of the ROS defense system, results in the relatively high vulnerability of β-cells to oxidative stress damage [39]–[40]. Mitochondria are important organelles for the generation of cellular energy and required for normal physiological functions. It has been indicted that mitochondria are very sensitive to oxidative stress, and disruption of mitochondrial function by mutated mitochondrial DNA is known to cause β-cell dysfunction and death that leads to an insulin-deficient form of type 1 DM [22], [40]. Recent studies have reported that toxic metals can induce apoptosis in several types of cells by causing mitochondrial dysfunction (disrupting MMP and promoting the release of mitochondria death effectors, such as cytochrome *c*, AIP, and EndoG from the intermembrane space of mitochondria into the cytosol) and activating caspase cascade signals [33], [41]. The results of our study showed that Cd induced ROS generation in RIN-m5F cells that led to apoptosis through loss of MMP and increased cytochrome *c* release. We also observed a significant increase in the 89-kDa cleaved form of PARP and subsequent activation of caspase-3, -7, and -9 proteases that was accompanied by a marked increase in intracellular Cd accumulation. These Cd-induced responses could be effectively reversed by NAC. In addition, the Bcl-2 family of proteins, which includes a balance of anti- and pro-apoptotic members involved in determining the fate of a cell lives or dies, has been demonstrated to induce the release of mitochondria death effectors, such as cytochrome *c* and the onset of mitochondrial dysfunction that subsequently regulates apoptosis pathways [42]–[43]. The tumor suppressor p53 acts as an upstream effector of Bcl-2 family members and is also an important activator for the intrinsic apoptosis pathway that is induced by ROS [44]–[45]. Here, we demonstrated that exposure of RIN-m5F cells to Cd resulted in an increase in *p53* and a reduction in *Bcl-2* gene expression, but had not effect on *Bax*, suggesting that p53 activation and the concomitant changes in the anti-apoptotic/pro-apoptotic ratio of Bcl-2 family members might contribute to the promotion of Cd-induced apoptosis. Therefore, these findings indicate that Cd-induced oxidative stress results in pancreatic β-cell death through the mitochondria-dependent apoptosis pathway.

MAPKs comprise a family of related ubiquitous proline-directed, protein-serine/threonine kinases, including JNK, ERK, and p38, that play an important role in the sequential transduction of biological signals from the cell membrane to the nucleus and regulate the many cellular responses to environmental stimuli, such as cell survival, transformation, and apoptosis [23], [24]. Recently, it has been indicated that activation of MAPK signaling pathways, which can be induced by oxidative stress via exposure to many injurious agents, contribute to the pathogenesis of neurodegenerative diseases through the induction of neuronal cell apoptosis [25], [46]. Oxidative stress, elicited by ROS, is a trigger for cell death and affects many pathophysiological processes. It is also implicated in human diseases, including cardiovascular diseases, neurodegenerative diseases, rheumatoid arthritis, and DM [30], [47]. A number of studies have indicated that the activation of inappropriate MAPK signaling pathways, which are induced by oxidative stress, contributes to pancreatic β-cells dysfunction and development of DM [39], [48]. Activation of the JNK- and/or p38 MAPK-mediated apoptosis signaling pathways involved in oxidative stress-induced death of pancreatic β-cells has been reported [26]–[27]. However, there is still a debate as to whether oxidative stress and MAPKs are the cause or the result of DM, and that is largely due to a lack of understanding of the detailed mechanisms by which oxidative stress and MAPK pathways function in pancreatic β-cell injury and the development of DM. The results of the present work found that Cd significantly increased phosphorylation of MAPK members including JNK, ERK, and p38 MAPK in RIN-m5F cells, which could be reversed by the antioxidant NAC. Pretreatment of cells with the specific JNK inhibitor SP600125 or JNK-specific si-RNA transfection, but not the ERK inhibitor PD98059 or p38 inhibitor SB203580, effectively suppressed Cd-induced cytotoxicity, apoptotic events (prevented, loss of MMP, PARP cleaved, and caspase-3 and caspase-7 activation), and blocked JNK protein activation. Furthermore, ROS generation was effectively decreased by pretreatment with NAC, but not SP600125 or JNK-specific si-RNA transfection, in Cd-exposed cells. These results indicate that oxidative stress-mediated signals are critical for downstream JNK activation-regulated mitochondria-dependent apoptosis pathway in Cd-induced pancreatic β-cell death.

Cd is widely used in industry and manufacturing, which is an important source of environmental contamination in human. To distinguish from occupational worker exposure, the two main sources of Cd exposure in people are diet and cigarette smoking [1], [49]. Cd content in human can gradually increase by exposure to Cd-contaminated foods, which is through bio-accumulated effects raising Cd levels in the food chain (such as 0.06 mg/L vs. 0.42–0.63 mg/L of Cd in rice from non-vs. Cd-contaminated areas), and may cause a higher incidence of many Cd-related diseases, including renal dysfunction, hepatotoxicity, osteoporosis, and cancers [4], [6], [49]. Notably, results of some epidemiological studies indicated significantly higher blood and urine levels of Cd in DM patients compared to healthy individuals [14]–[16]. It has also been revealed a significantly positive correlation between increases in urinary Cd and prevalence of impaired fasting glucose levels (odds ratio [OR]: 1.48–2.05, 95% confidence interval [CI]: 1.21–2.95) and DM (OR: 1.24–1.45, 95% CI: 1.06–1.97) [17]. Moreover, in sub- or chronic investigations, a marked increase in blood glucose levels, glucose intolerance, gluconeogenic enzymes, and even enhanced Cd toxicity in diabetic conditions, after rats exposed to Cd (0.5–2 mg/kg/day) was developed, which was accompanied with significant Cd accumulation in the blood and/or pancreas [11]–[12], [50]–[51]. It has been reported that chronic exposure of neonatal rats to Cd (1 mg/kg/day) significantly disturbed glucose homeostasis and destroyed pancreatic islets insulin secretion as reflected by the development of hyperglycemia [52]. Although some animal studies have indicated a possible link between Cd exposure and blood glucose unbalance and/or pancreatic islets dysfunction, there is no literature to explore the possible mechanisms underlying pancreatic islets dysfunction and injuries caused by Cd *in vivo*. Here, our present results showed that male mice after exposure to CdCl_2_ (0.5 and 1.0 mg/kg/day) for 6 consecutive weeks had significantly decreased in plasma insulin levels and induced in the abnormalities of glucose tolerance, which was accompanied with marked Cd accumulation in the blood (1.31±0.11–9.46±2.46 μg/L (approximately 0.01–0.08 μM)) and pancreas (26.80±3.76–339.0±19.4 ng/g w.t.). Moreover, plasma MDA levels (as an oxidative stress damage marker) were increased in Cd-treated mice. Determination of islet cells apoptosis also markedly revealed an increase in TUNEL-positive cells in Cd-exposed mice. These Cd-induced responses could be effectively reversed by NAC. On the basis of these results implicate that Cd-induced oxidative stress damage plays an important role in pancreatic islet β-cell dysfunction and apoptosis.

**Figure 8:**
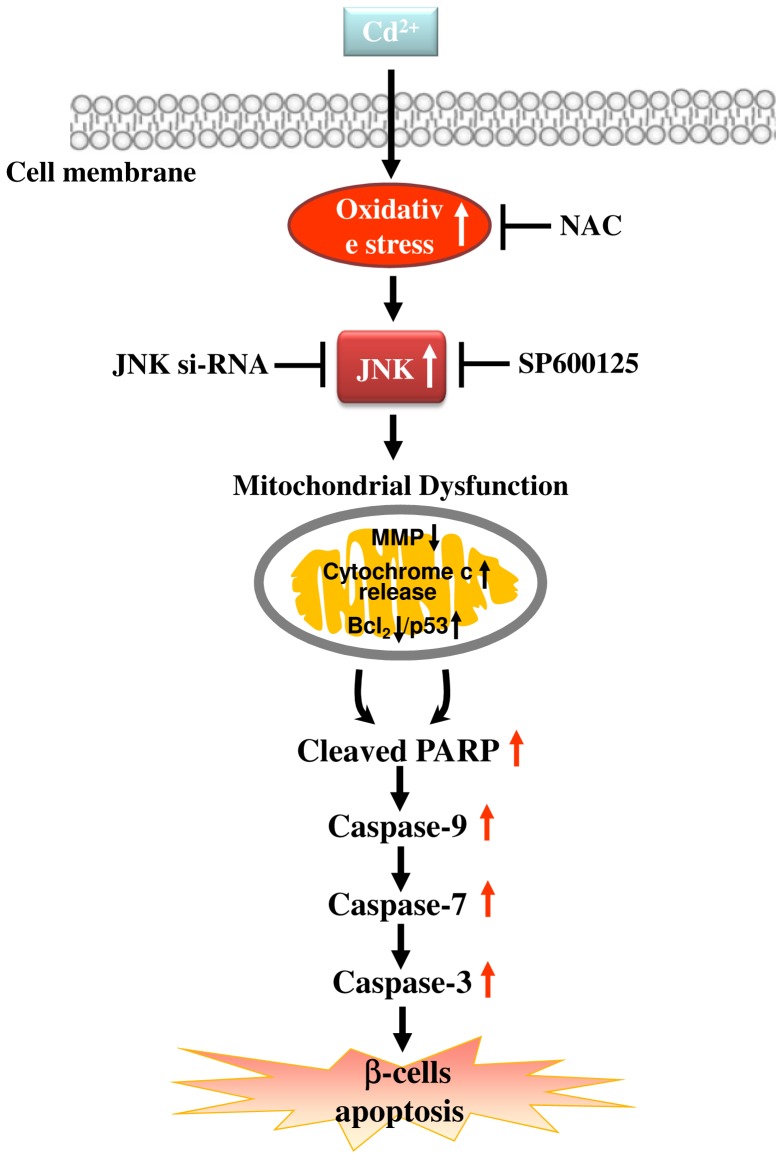
Schematic diagram of the signaling pathways involved in Cd-induced pancreatic β-cell apoptosis. Proposed models representing that Cd induces pancreatic β-cell apoptosis through ROS triggering JNK activation-regulated mitochondria-dependent apoptotic signaling cascades are illustrated in this schematic diagram.

In conclusion, this study shows that Cd is capable of inducing oxidative stress damage that causes suppression of insulin secretion and apoptosis in pancreatic islet β-cells *in vivo* and *in vitro*. Furthermore, as shown in Fig. 8, the results of this study indicated that Cd induces pancreatic β-cell death through the mitochondria-dependent apoptosis pathway, where mitochondrial dysfunction (loss of MMP, increase in cytochrome *c* release, and decrease in *Bcl-2* and increase in *p53* expression), increased PARP cleavage, and activation of caspase-3/-7-/9 are critical events for apoptosis. This study is the first report to demonstrate that oxidative stress regulates downstream JNK activation, which is the pivotal signaling event in Cd-induced pancreatic β-cell apoptosis. Therefore, our research indicates that oxidative stress-regulated JNK activation may be a target molecule for Cd-induced pancreatic β-cell injuries, and also provide further evidence to confirm the possible role of Cd as an environmental risk factor for diabetes.

## Supporting Information

Figure S1 **Influence of body weight changes in Cd-exposed mice.** Mice were orally gavaged with 0.5 or 1 mg kg^−1^ day^−1^ CdCl_2_ for 6 consecutive weeks, and body weight changes were recorded for every week. Data are presented as mean ± S.D.; *n* = 15. *p<0.05 as compared with vehicle control.
(TIF)

Figure S2 **Plasma insulin secretion, glucose tolerance test, plasma lipid peroxidation production and islet cells apoptosis in Cd-exposed mice.** Mice were orally gavaged with 0.5 or 1 mg kg^−1^ day^−1^ CdCl_2_ for 6 consecutive weeks in the presence or absence of NAC (150 mg kg^−1^ day^−1^, oral application by gavage), and (A) plasma insulin secretion was detected by insulin assay ELISA kit, (B) oral glucose tolerance tests (a) and insulin in fasting mice (after 1 g/kg glucose loading for 30 min) were carried out in mice given distilled water (vehicle) or Cd for 6 consecutive weeks and determined, (C) plasma malondialdehyde (MDA) levels in vehicle or Cd-exposed mice were examined by using the lipid peroxidation assay kit as described in the Materials and Methods section, and (D) apoptosis of pancreatic islet cells was determined by TUNEL assay. Data in A–C are presented as mean ± S.D.; *n* = 15. *p<0.05 as compared with vehicle control. **^#^** *p*<0.05 as compared with Cd alone.
(TIF)

Table S1 **Whole blood cadmium levels in Cd-exposed mice.**
(DOC)

Table S2 **Cadmium levels of pancreas in Cd-exposed mice.**
(DOC)
