# Supplementary figures and images for: Cadmium Induces Apoptosis in Pancreatic β-Cells through a Mitochondria-Dependent Pathway: The Role of Oxidative Stress-Mediated c-Jun N-Terminal Kinase Activation
Source: PLoS One. 2013 Feb 6;8(2):e54374. doi: 10.1371/journal.pone.0054374 (PMC3566170; doi:10.1371/journal.pone.0054374)

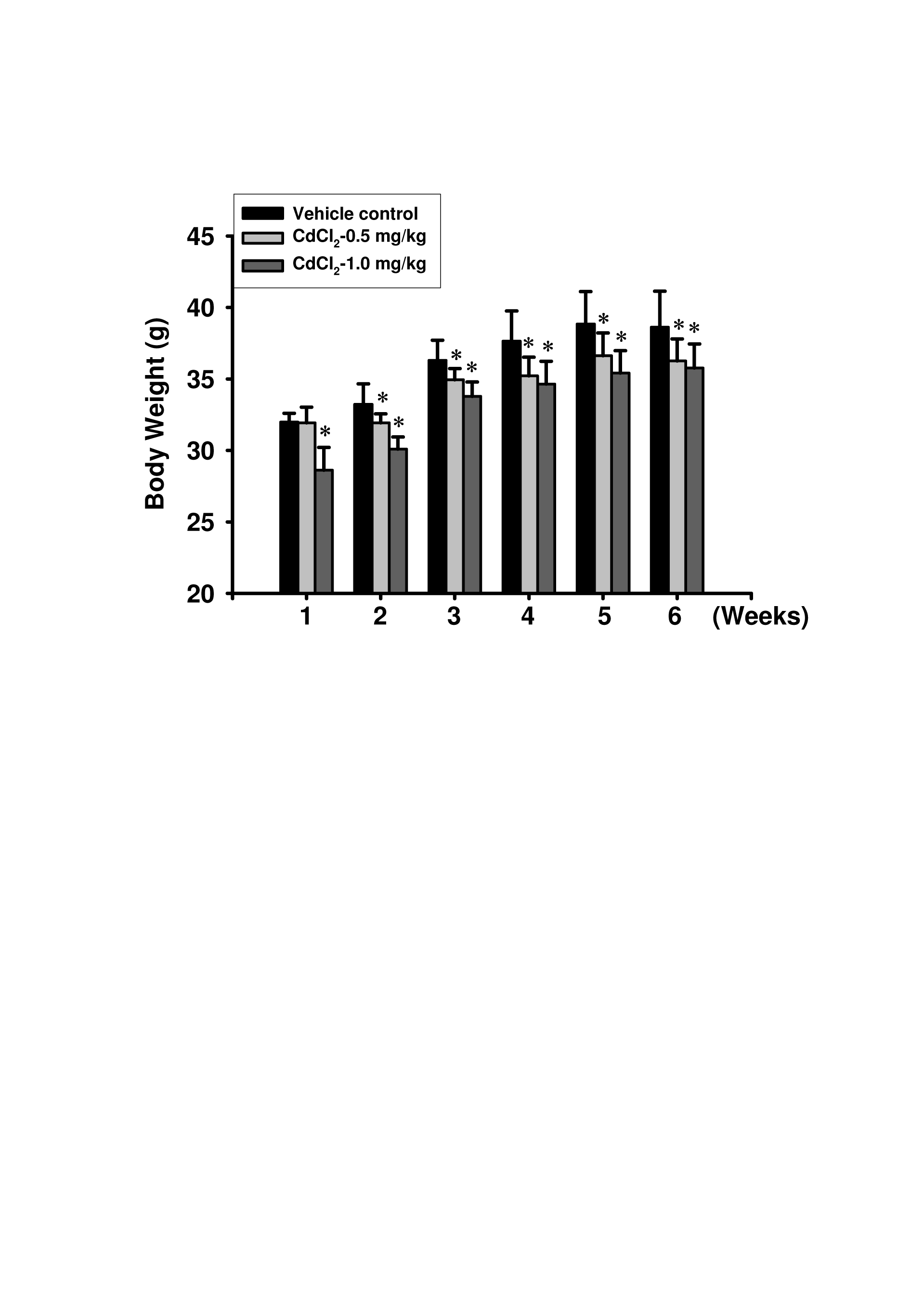

Supplement: Figure S1 — Influence of body weight changes in Cd-exposed mice. Mice were orally gavaged with 0.5 or 1 mg kg−1 day−1 CdCl2 for 6 consecutive weeks, and body weight changes were recorded for every week. Data are presented as mean ± S.D.; n = 15. *p<0.05 as compared with vehicle control. (TIF) [file pone.0054374.s001.tif]

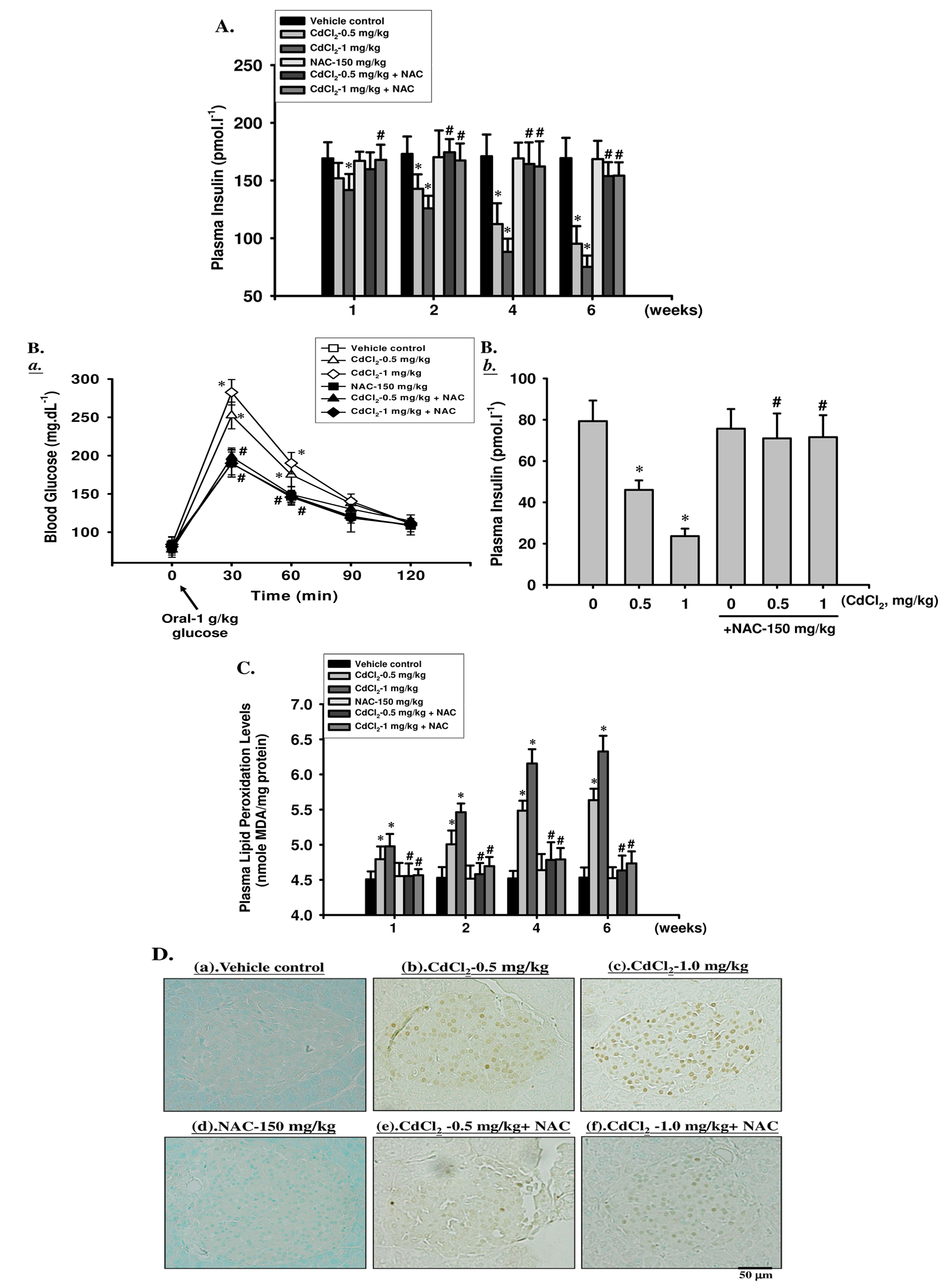

Supplement: Figure S2 — Plasma insulin secretion, glucose tolerance test, plasma lipid peroxidation production and islet cells apoptosis in Cd-exposed mice. Mice were orally gavaged with 0.5 or 1 mg kg−1 day−1 CdCl2 for 6 consecutive weeks in the presence or absence of NAC (150 mg kg−1 day−1, oral application by gavage), and (A) plasma insulin secretion was detected by insulin assay ELISA kit, (B) oral glucose tolerance tests (a) and insulin in fasting mice (after 1 g/kg glucose loading for 30 min) were carried out in mice given distilled water (vehicle) or Cd for 6 consecutive weeks and determined, (C) plasma malondialdehyde (MDA) levels in vehicle or Cd-exposed mice were examined by using the lipid peroxidation assay kit as described in the Materials and Methods section, and (D) apoptosis of pancreatic islet cells was determined by TUNEL assay. Data in A–C are presented as mean ± S.D.; n = 15. *p<0.05 as compared with vehicle control. # p<0.05 as compared with Cd alone. (TIF) [file pone.0054374.s002.tif]
