# Supplementary material for: Cadmium Induces Apoptosis in Pancreatic β-Cells through a Mitochondria-Dependent Pathway: The Role of Oxidative Stress-Mediated c-Jun N-Terminal Kinase Activation
Source: PLoS One. 2013 Feb 6;8(2):e54374. doi: 10.1371/journal.pone.0054374 (PMC3566170; doi:10.1371/journal.pone.0054374)
Supplement: Table S1 — Whole blood cadmium levels in Cd-exposed mice. (DOC) [file pone.0054374.s003.doc]

**Table S1.** Whole blood cadmium levels in Cd-exposed mice.

Group

Weeks Vehicle control CdCl_2_-0.5 mg/kg CdCl_2_-1 mg/kg

1 0.25 ± 0.05 1.31 ± 0.11**^*^** 3.64 ± 0.49**^*^**

2 0.28 ± 0.07 1.46 ± 0.20**^*^** 3.86 ± 0.98**^*^**

4 0.31 ± 0.08 2.47 ± 0.54**^*^** 6.75 ± 1.91**^*^**

6 0.41 ± 0.05 2.98 ± 0.56**^*^** 9.46 ± 2.45**^*^**

1. Cadmium content of whole blood was expressed as μg/L.
2. Data were presented as mean ± S.D. (*n* = 15 for each group). **^*^***p* < 0.05 as compared with the vehicle control group.
