# Supplementary material for: Cadmium Induces Apoptosis in Pancreatic β-Cells through a Mitochondria-Dependent Pathway: The Role of Oxidative Stress-Mediated c-Jun N-Terminal Kinase Activation
Source: PLoS One. 2013 Feb 6;8(2):e54374. doi: 10.1371/journal.pone.0054374 (PMC3566170; doi:10.1371/journal.pone.0054374)
Supplement: Table S2 — Cadmium levels of pancreas in Cd-exposed mice. (DOC) [file pone.0054374.s004.doc]

**Table S2.** Cadmium levels of pancreas in Cd-exposed mice.

Group

Weeks Vehicle control CdCl_2_-0.5 mg/kg CdCl_2_-1 mg/kg

1 5.33 ± 0.51 26.80 ± 3.76**^*^** 78.40 ± 12.7**^*^**

2 5.13 ± 0.35 34.40 ± 7.44**^*^** 90.80 ± 13.7**^*^**

4 2.33 ± 0.57 78.60 ± 18.2**^*^** 198.0 ± 20.9**^*^**

6 4.47 ± 0.76 149.0 ± 14.8**^*^** 339.0 ± 19.4**^*^**

1. Cadmium content of pancreas tissue was expressed as ng/g w.t..
2. Data were presented as mean ± S.D. (*n* = 15 for each group). **^*^***p* < 0.05 as compared with the vehicle control group.
